# Room temperature multi-phonon upconversion photoluminescence in monolayer semiconductor WS_2_

**DOI:** 10.1038/s41467-018-07994-1

**Published:** 2019-01-10

**Authors:** J. Jadczak, L. Bryja, J. Kutrowska-Girzycka, P. Kapuściński, M. Bieniek, Y.-S. Huang, P. Hawrylak

**Affiliations:** 1 Department of Experimental Physics, Wroclaw University of Science and Technology, Wroclaw, 50-370 Poland; 2 Department of Physics, University of Ottawa, Ottawa, K1N 6N5 Ontario, Canada; 3 Department of Electronic Engineering, National Taiwan University of Science and Technology, Taipei, 106 Taiwan; 4 Department of Theoretical Physics, Wrocław University of Science and Technology, Wybrzeże Wyspiańskiego 27, 50-370 Wroclaw, Poland

## Abstract

Photon upconversion is an anti-Stokes process in which an absorption of a photon leads to a reemission of a photon at an energy higher than the excitation energy. The upconversion photoemission has been already demonstrated in rare earth atoms in glasses, semiconductor quantum wells, nanobelts, carbon nanotubes and atomically thin semiconductors. Here, we demonstrate a room temperature upconversion photoluminescence process in a monolayer semiconductor WS_2_, with energy gain up to 150 meV. We attribute this process to transitions involving trions and many phonons and free exciton complexes. These results are very promising for energy harvesting, laser refrigeration and optoelectronics at the nanoscale.

## Abstract (web summary)

Two-dimensional materials show promise for photon upconversion processes due to their strong photon-exciton and phonon-exciton interactions. Here, the authors report room temperature upconversion photoluminescence in monolayer WS_2_ with high energy gain up to 150 meV based on variable temperature.

## Introduction

Photon upconversion is an anti-Stokes process in which an absorption of a photon leads to a reemission of a photon at energy higher than the excitation energy. The upconversion photoemission has been already demonstrated in rare earth atoms in glasses^1–3^, semiconductor quantum wells^4–7^, nanobelts^8^, carbon nanotubes^9^ and atomically thin semiconductors^10,11^ at cryogenic temperatures. The atomically thin semiconductors based on transition metal dichalcogenides are particularly promising for room temperature upconversion due to their very strong photon–exciton^12–32^ and phonon–exciton interactions^14^.

Recently, Jones et al.^10^ reported phonon-mediated upconversion of photon emission by ~30 meV up to 250 K, from trion (X^−^) to exciton (X), in a single layer of WSe_2_. This was possible because the strong confinement of carriers to a single layer results in a very high binding energy of an electron to an exciton forming a trion^14–23,25^, ~30 meV, comparable with a phonon energy.

Here, we demonstrate a room temperature upconversion photoluminescence process in a monolayer semiconductor WS_2_ with a larger energy gain, up to 150 meV. We also show that the energy gain significantly depends on the temperature and increases from 42 meV at 7 K to 150 meV at 295 K.

## Results

### Room temperature robust photon upconversion by 150 meV

**Fig. 1:**
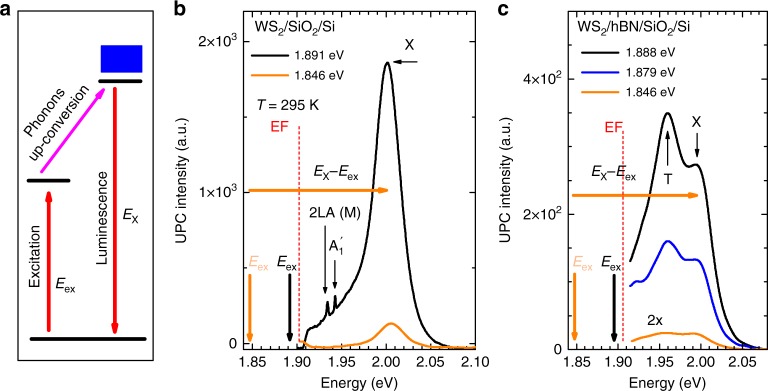
Upconversion photoemission process in monolayer WS_2_. **a** The schematic representation of upconversion process. **b** Examples of the upconversion photoemission spectra in WS_2_/SiO_2_/Si recorded for different excitation photon energies. **c** Examples of the upconversion photoemission spectra in WS_2_/hBN(136 nm)/SiO_2_/Si detected for different excitation photon energies. To reduce laser scattering light short-pass (in wavelength) edge filters (EF) with edge at 652 nm (1.90 eV) were used

Figure 1a–c shows the main result of this work, demonstrating a robust upconversion of excited light. Figure 1a shows a schematic representation of our experiment. Photons from exciting laser field E_ex_ couple the ground state to excited states with energy below the bandgap of a semiconductor^7,9^. Then the electrons in an excited state absorb phonons and are transferred to a higher energy excitonic state X, from which they recombine to the ground state, emitting a photon at an energy higher than the exciting laser photon energy. Fig. 1b demonstrates upconversion of exciting laser photons in an atomically thin semiconductor WS_2_. A single layer of WS_2_ is deposited on a commonly used SiO_2_/Si substrate, and emission spectra are recorded in ambient environment and at room temperature of 295 K. We excite with a laser having photon energy E_ex_ = 1.85 eV, indicated with an orange arrow, and observe emission at much higher energy, centred on a free exciton energy E_X_ ≈ 2.008 eV. In Figure 1b, we clearly see that the incident photons are converted into emitted photons with an energy higher by 150 meV, which is about five times more than previously observed in ref. ^10^. For comparison, Fig. 1c demonstrates the effect of an extra hBN layer deposited between SiO_2_/Si and WS_2_ on the upconversion photoluminescence (UPC PL) process. It is clearly seen that exciting photons are also converted into emitted photons with energy higher by about 150 meV, even though the intensity of the upconversion emission detected in WS_2_/hBN/SiO_2_/Si heterostructure is one order of magnitude lower than in WS_2_ deposited directly on the SiO_2_/Si substrate. The intensity of emitted upconverted photons in both WS_2_/SiO_2_/Si and WS_2_/hBN/SiO_2_/Si heterostructures strongly correlates with the intensity of normal photoluminescence (PL), spectral shape of which strongly depends on the thickness of hBN layer. This is why, for further studies of the excitonic upconversion photoluminescence in ambient at 295 K, we choose single layers of WS_2_ exfoliated on SiO_2_/Si substrate, as they exhibit the most prominent emission attributed to the neutral exciton (X).

**Fig. 2:**
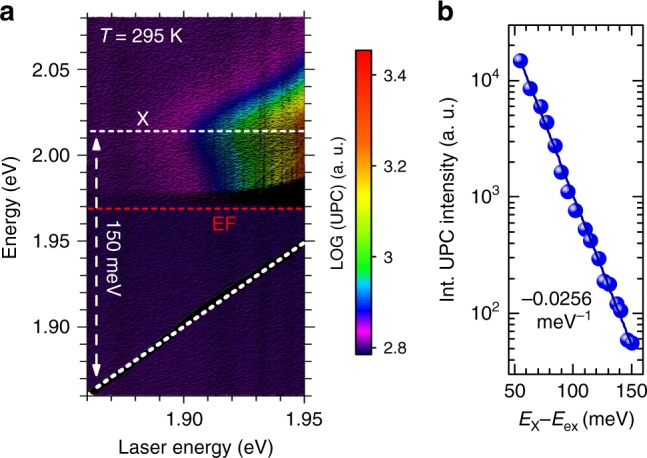
Excitation energy dependence of the upconversion photoemission. **a** Evolution of the upconversion emission spectra as a function of the laser excitation photon energy in WS_2_/SiO_2_/Si, recorded in ambient, at 295 K. **b** The dependence of the upconversion integrated intensity on the energy difference of the neutral exciton (X) and the excitation photon energy (E_X_–E_ex_)

Let us now fully characterize the upconversion photoluminescence process, i.e., variations in the upconverted photoemission spectrum as a function of an incident photon energy E_ex_. In this experiment, a short-pass edge filter (EF), with the edge at 632 nm (1.97 eV), was used. For all exciting photon energies E_ex_ one line at ~2.008 eV, related to the neutral exciton X emission, is observed in the spectra. The upconversion photoluminescence intensity of this line strongly depends on the excitation photon energy, whereas spectral shape and peak position remain nearly unchanged upon variation of the excitation energy, which is clearly seen in Fig. 2a. Figure 2a shows the colour map of the upconversion photoemission intensity as a function of the exciting photon energy. The exciting photon energy required to achieve a detectable upconversion photoluminescence (gradual intensity growth just above the noise floor) is found to exceed E_ex_ = 1.85 eV. Fig. 2b presents the dependence of the upconversion photoluminescence integrated intensity in the spectral range from 1.91 eV to 2.10 eV on the energy difference (E_X_–E_ex_) of the exciton X and the exciting photon energy E_ex_. For the energy difference E_X_–E_ex_ from 50 meV to 155 meV, the integrated upconversion photoluminescence intensity decreases with decreasing excitation photon energy with the rate equal to −0.0256 meV^−1^.

### Identifying the upconversion photoluminescence mechanism

**Fig. 3:**
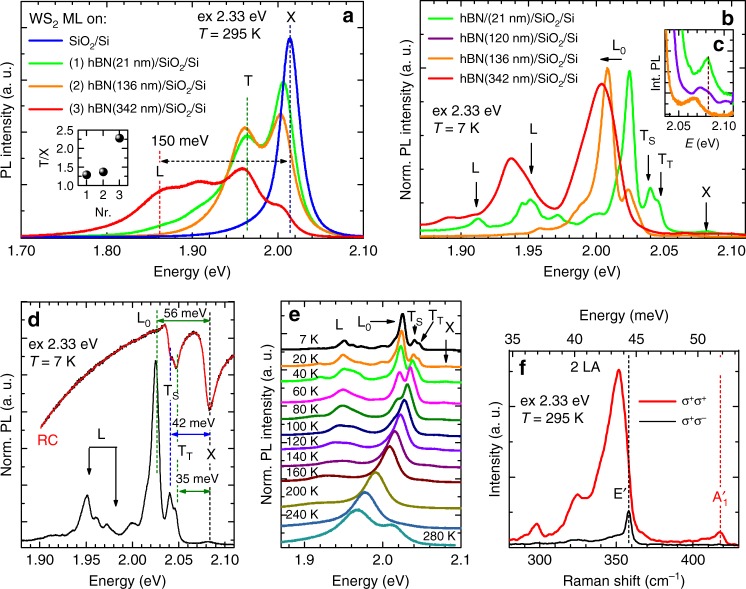
Photoluminescence, reflectance contrast and Raman scattering. **a** Examples of photoluminescence spectra of monolayer WS_2_ deposited on SiO_2_/Si substrate and on hBN/SiO_2_/Si substrates with different thicknesses of hBN layers, excited with incident photon energies of 2.33 eV (532 nm), and recorded in ambient at 295 K. The inset shows the T/X emission intensity ratio for different flakes, 1, 2 and 3, respectively. **b** Examples of photoluminescence spectra of monolayer WS_2_ deposited on hBN/SiO_2_/Si substrates with different thicknesses of hBN layers, excited with incident photon energies of 2.33 eV (532 nm), and recorded in vacuum at 7 K. **c** The red-shift of neutral exciton (X) emission line for increasing thickness of hBN layer. **d** The comparison of low temperature PL and RC spectra recorded in WS_2_/hBN(21 nm)/SiO_2_/Si. **e** The temperature evolution of PL spectra in WS_2_/hBN(21 nm)/SiO_2_/Si. **f** Helicity resolved Raman spectra excited with 532 nm laser line for WS_2_/SiO_2_/Si

We now turn to identifying mechanism responsible for the upconversion photoluminescence. Figure 3a shows photoluminescence spectra excited with laser having photon energy of 2.33 eV (532 nm). The spectra were measured in ambient at 295 K for monolayer WS_2_ deposited on the SiO_2_/Si substrate or hBN/SiO_2_/Si substrates with different thicknesses of hBN layers (21 nm, 136 nm, 342 nm), respectively. In the PL spectrum of the WS_2_/SiO_2_/Si (blue line), we distinguish only one, dominant, spectral line at 2.014 eV. In comparison with previous studies, including ours^17–21^, we attribute this line to the free exciton X emission. For all the WS_2_/hBN/SiO_2_/Si heterostructures with finite hBN thickness, we observe X line redshifting to 2.006 eV, about 8 meV below its position recorded for the WS_2_ monolayer deposited on SiO_2_/Si. This energy difference is related to the change of the exciton-binding energy E_b_ and the bandgap energy E_g_ (E_X_ = E_g_ − E_b_). The different dielectric environments of WS_2_ monolayers lead to the reduction of both E_g_ and E_b_ energies in WS_2_/hBN/SiO_2_/Si heterostructures in comparison with the WS_2_/SiO_2_/Si structures. Moreover, additional emission lines appear in the emission spectra of WS_2_/hBN/SiO_2_/Si structures. One of the lines, positioned at the energy of 1.964 eV, 42 meV below that of the exciton (X), is attributed to a negatively charged exciton, trion (T), whereas the lower-energy broad features labelled as L, positioned at ~1.86 meV, are attributed to strongly localized excitons^20,21^. The L lines in the PL of the WS_2_/hBN/SiO_2_/Si heterostructures are recorded about 150 meV below that of the free exciton. These results show that an extra hBN layer used between the flake and SiO_2_/Si substrate can act as a buffer layer^33^ and changes the doping level in the monolayer system. Here, it is manifested by the altering trion to exciton emission intensity ratio (T/X) shown in the inset of Fig. 3a. The X and T emission intensities are obtained by the fitting of the PL spectra by Lorentz function in spectral range from 1.7 eV to 2.10 eV. For normal WS_2_/SiO_2_/Si structure, the trion peak is hardly detected in the PL spectra, whereas for WS_2_/hBN/SiO_2_/Si heterostructures with increasing hBN thicknesses the T/X PL intensity ratio increases. The rising value of the T/X PL intensity ratio indicates that the increasing of hBN layer thickness leads to the growth of two-dimensional electron gas concentration in monolayer WS_2_. This dependence can be explained by the fact that the WS_2_ is naturally n-doped whereas positively charged defects (or charge inhomogeneity) are embedded in SiO_2_ surface, close to the monolayer WS_2_. Hence, the positively charge defects in SiO_2_ surface strongly influence an electron charge in WS_2_. Intuitively, the electron-binding energy to the charged defect embedded in SiO_2_ is the largest for WS_2_/SiO_2_/Si structures and should decrease with increasing thickness of hBN layer. Our experimental results are qualitatively consistent with recent theoretical calculations of the ground-state energies of electrons as a function of the distance of a positive point-charge defect from the mid-plane of the monolayer^34^. Namely, Tuan et al.^34^ have shown that the electron-binding energy is the largest when the defect is at the surface, and it significantly decreases from 150 meV to a few meV as the distance between point-charge defect and the mid-plane of the monolayer exceeds 100 nm. Interestingly, the L lines attributed to localized excitons emerge in the PL spectra on the low energy wing of the trion when hBN layer is placed between the WS_2_ monolayer and SiO_2_ substrate and they are well resolved in the spectra and comparable with the trion emission for hBN thickness higher than 300 nm (Fig. 3a). when the contact with extrinsic defects in SiO_2_ is reduced.

In order to get further inside into the nature of observed excitonic complexes, we perform complementary temperature-dependent PL measurements. Figure 3b compares low temperature (7 K) spectra, normalized to maximum photoluminescence intensity, detected for selected WS_2_/hBN/SiO_2_/Si heterostructures with different thicknesses of hBN layers (21 nm, 120 nm, 136 nm), respectively. There is a qualitative correlation between the energy of the peak associated with particular transition and the thickness of the hBN layer. The emission lines detected at higher energies, labelled as X, T_S_, T_T_ and L_0_, exhibit an apparent red shift with increasing thickness of hBN (for X line see Fig. 3c), whereas the series of lower energy L lines remains energetically inert. Based on this observation, we divide all observed optical transitions into two groups. The first group is associated with the nearly free states of the neutral exciton (X), intravalley spin singlet trion (T_S_)^10,19–21^, intervalley spin triplet trion (T_T_)^10,19–21^ and intervalley singlet trion (L_0_)^20,21^, respectively. The second group is attributed to the strongly localized states, likely excitons bound to donors or acceptors. Donor or acceptor levels likely originate from vacancies, which are randomly created during mechanical exfoliation, therefore the emission intensity of bound excitons differs slightly from flake to flake due to different amount and types of created vacancies and the difference in the local two-dimensional electron gas concentration. We have observed the effects related to inhomogeneous two- dimensional electron gas concentration in monolayer WS_2_ in our previous studies^21^, where we have found a correlation between the strength of the trion and exciton resonances in RC spectra recorded at different points on the monolayer. Moreover, the L states are strongly localized in the gap, hence they are weakly influenced by the change of the dielectric environment and remain energetically inert. These optical transitions also do not play a significant role in absorption type experiments, such as reflectance contrast (RC) spectroscopy. Figure 3d compares the photoluminescence and reflectivity contrast spectra recorded at T = 7 K for the WS_2_/hBN(21 nm)/SiO_2_/Si structure. It is clearly seen that in the reflectivity contrast measurements, we probe only nearly free states (X, T_T_, T_S_ and L_0_), whereas L series is not detected. On the other hand, the L peaks are clearly seen in the PL spectra. The reason for the difference in the strength of optical amplitude of the different excitonic transitions in PL and RC spectra is that the strength of excitons resonances in reflectivity is determined by respective density of states, whereas the PL intensity is contributed additionally by a state occupation factor.

Finally, let us analyse the temperature evolution of photoluminescence spectra detected for WS_2_/hBN(21 nm)/SiO_2_/Si structure, which are presented in Fig. 3e. We see that the L series significantly decays above 160 K. It can be explained by the fact that the excitons gain kinetic energy and become less susceptible to the capture process. For different WS_2_/hBN/SiO_2_/Si samples (not presented here), this effect occurs for slightly higher or lower temperatures and depends on the thickness of the hBN layer. Also, we observe that the energy position of the L series does not change as clearly as for nearly free states, which rapidly approach localized exciton energies with increasing temperature.

We now turn to identifying potential phonon modes involved in the upconversion process. Figure 3f presents helicity resolved Raman scattering spectra of a monolayer WS_2_ deposited on an SiO_2_/Si substrate excited with the 532 nm laser line with σ^+^ polarized excitation and detection either in σ^+^ or σ^−^ polarization, labelled σ^+^σ^+^ or σ^+^σ^−^. This method allows us to resolve and precisely assign degenerate Raman bands for the monolayer WS_2_^35^. The first order out-of-plane A’_1_ mode is visible only in σ^+^σ^+^ configuration, whereas the in-plane E’ is detected only in the spectra of opposite helicity σ^+^σ^−^. The Raman spectra clearly identify the E’ and A’_1_ optical phonons with energies ~44 meV (357 cm^−1^) and ~52 meV (418 cm^−1^), respectively. In the upconversion photoluminescence process, the A_2_’’ vibration at 441 cm^−1^ (~55 meV)^36^ can be also considered. This phonon is Raman inactive in bulk, but becomes partially active for one or a few layers under resonant excitation^37^.

Jones et al.^10^ have attributed the upconversion photoluminescence process from a negatively charged exciton to a neutral exciton resonance in monolayer WSe_2_, producing spontaneous anti-Stokes emission with an energy gain of 30 meV, to doubly resonant Raman scattering mediated by the A’_1_ optical phonon. They based their interpretation on detailed polarization resolved experiments and the fact that the 30 meV separation between neutral and charged excitons coincides with the A’_1_ energy equal to 31 meV. They observed preservation of valley polarization for excitation both above and below X, consistent with the polarization properties of the A’_1_ phonon. In contrast, the E’ mode in Raman spectra from WSe_2_, is cross-polarized and unpolarized for circularly and linearly polarized excitation, respectively. Additionally, the symmetry of the electronic states within the K valleys dictates a stronger interaction with the out-of-plane A’_1_ phonon than with the in-plane E’ mode^38,39^.

We observe the same (as in ref. ^10^) polarization-dependent relation between the normal and upconversion photoluminescence and the A’_1_ optical phonon in Raman spectra. In contrast to ref. ^10^, where the upconversion photoluminescence process is mediated by one A’_1_ phonon, in our experiment more phonons are involved in the upconversion process. At room temperature the energy gain of 150 meV in the upconversion photoluminescence process in monolayer WS_2_ closely matches the energy of three A’_1_ phonons equals to 156 meV. While, the three A’_1_ phonon-mediated upconversion of photon emission by 150 meV is most probable, other combinations of optical phonons, A’_1_, E’ and A_2_’’, in the observed upconversion photoluminescence process should be also considered. For example, in the recent work Tuan et al.^34^ have proposed a model of phonon-assisted recombination facilitated by virtual trion states.

What is the origin of the initial states in the upconversion photoluminescence process? One of the possible explanation is that incident photon is in resonance with an exciton localized on impurity. This assignment of the initial upconversion state seems to be reasonable due to the proximity of localized states with the lowest excitation energies (~150 meV). However, in pseudo-absorption reflectivity contrast experiment we do not observe any features at energies specific to excitons localized on impurities. While this could be explained due to significant distribution of different impurity states, absence of any features as observed in, e.g., L band, shows that the absorption on excitons localized on impurities is week. In contrast, we observe the exponential drop of upconversion intensity as excitation energy is lowered from the exciton into the energy gap (see Fig. 2). Hence, similarly as in ref. ^10^, we attribute the observed upconversion photoluminescence to the spontaneous excitonic anti-Stokes Raman scattering process, where the incident and emitting photons are in resonance with the trion and neutral exciton, respectively.

In contrast to the observations of Jones et al.^10^ where in the upconversion photoluminescence from the charged to the neutral exciton one phonon was involved, in our experiments the upconversion is related to multi-phonon process. The absorption of an incident photons is related to the states from the tail of the trion (see Fig. 3a). Moreover, in our previous studies^21^ we have observed that the absorption features broaden significantly as temperature increases, which leads to the increase of the absorption from the trion tail. Also, as it will be shown below, we detect the maximum of upconversion intensity for the excitation energy equal to the trion energy. Further experimental and theoretical studies are planned to elucidate the upconversion mechanism.

### Power and carrier density dependence of upconversion spectrum

**Fig. 4:**
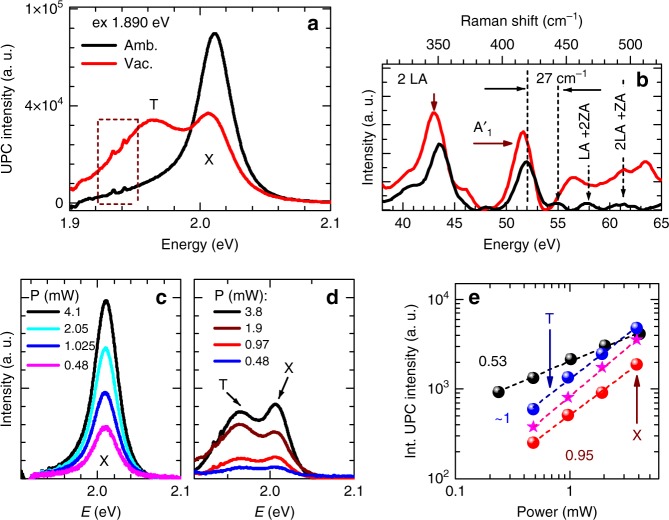
Excitation power dependence of the upconversion photoemission. **a** Comparison of the spectra in monolayer WS_2_ deposited on SiO_2_/Si substrate, recorded in ambient (black line) and vacuum (red line), excited with the same incident photon energy 1.890 eV and power, at 295 K. **b** Comparison of the anti-Stokes Raman spectra measured in ambient (black line) and vacuum (red line), obtained by subtracting the emission from the corresponding UPC spectra in **a**. **c**, **d** examples of spectra excited with different laser powers, recorded in ambient and vacuum, respectively. **e** The integrated upconversion photoemission intensity plotted as a function of excitation power density for total spectra recorded in ambient (black circles), in vacuum (magenta stars), for X peak in vacuum (red points), for T peak in vacuum (blue point)

To gain more insight into the character of the excitonic upconversion photoluminescence in monolayer WS_2_, we performed excitation power-dependent measurements, with the excitation photon energy of 1.890 eV, corresponding to the broad emission of the localized excitons L (see Fig. 4). The experiments were carried out on the WS_2_ monolayer deposited on SiO_2_/Si at room temperature, in both ambient and vacuum conditions. The two different conditions allow us to investigate carrier density dependence of the upconversion process. It is well established that under ambient conditions the physisorbed O_2_ and H_2_O molecules deplete n-type materials such as WS_2_ or MoS_2_, much more than conventional electric field gating^22,23^. This is also clearly manifested in Fig. 4a, where the spectral shape of lines strongly differs between experiments performed in ambient (black line) and vacuum (red lines). This confirms previous reports that in ambient, the two-dimensional electron gas (2DEG) concentration is strongly depleted^21^. The upconversion photoluminescence spectra of WS_2_ measured in ambient conditions are dominated by the exciton emission X, whereas the trion T is hardly detected in the spectra. In contrast, in vacuum the upconversion photoluminescence spectra clearly resolves emission of both the exciton and trion with comparable intensities. Their energy separation equals to 42 meV, and matches the values reported in previous studies^20,21^. Additionally, Fig. 4b shows the comparison of the anti-Stokes Raman spectra measured in ambient (black line) and vacuum (red line), obtained by subtracting a Lorentz fit of the upconversion emission from the total upconversion emission spectrum (Fig. 4a), where the Raman features are indicated by the dashed rectangle. The Raman scattering peaks seen in ambient at 352cm^−1^ and at 418 cm^−1^ are assigned to the 2 LA (M) and A’_1_ phonon modes, respectively. In vacuum, these phonons shift towards lower frequencies by 5 cm^−1^ and 2 cm^−1^, respectively. Moreover, in ambient, in the highest energy region we can also distinguish weak broad features at about 446 cm^−1^ (~55 meV), 470 cm^−1^ and 495 cm^−1^, similarly to work of Molas et al.^40^, who report resonant Raman scattering study of monolayer WS_2_ and propose identification of the peaks at 470 cm^−1^ and 495 cm^−1^ as the combined acoustic processes; LA (M) + 2ZA(M) and 2 LA(M) + ZA(M), respectively. The feature observed in our experiment at the energy ~27 cm^−1^ higher than the energy of the A’_1_ peak may be assigned to the partially active A’’_2_ phonon or it can result from multi-phonon processes, which involve principal optical phonons (E’ or A’) and additional acoustic phonons^40^.

Figure 4c and d presents typical photoemission spectra recorded for several excitation power densities, in ambient and in vacuum, respectively. Figure 4e shows the total upconversion intensity in ambient (black points) and vacuum (magenta stars) as a function of excitation power density, integrated over the same energy range from 1.9 eV to 2.1 eV. They reveal weak sublinear (0.53) and linear (~1) dependence for increasing excitation power in ambient and in vacuum, respectively. In addition, Fig. 4e presents power dependence of the X and T emission intensities obtained by fitting of the upconversion emission spectrum by Lorentz function in corresponding spectral range (1.9–2.1 eV). The X (red points) and T (blue points) emissions show nearly linear (0.95) and linear (~1) dependence, respectively. Interestingly, our observation clearly shows that upconversion mechanism strongly depends on 2DEG concentration. The weak sublinear dependence in ambient may be related to the changes of the charge on donor states^25,41^.

Nevertheless, we assume that both mechanisms originate from the multiple-phonon-assisted upconversion, since the threshold energy gain of ~150 meV indicates that the upconversion can be related to the Raman scattering process involving particular combination of the optical phonons. Moreover, for our experimental conditions, photons with the energy below the gap are sufficient to photo-ionize electrons from the donor level, which according to recent numerical calculation is positioned a few hundred meV below the conduction band^25^. Furthermore, as all the data of total upconversion intensities follow the sublinear and linear relationship, we exclude the possibility of nonlinear optical generation of the observed upconversion photoluminescence, such as two-photon excitation-induced emission^42,43^ and exciton Auger scattering^44,45^.

### Temperature dependence of the upconversion photoluminescence

**Fig. 5:**
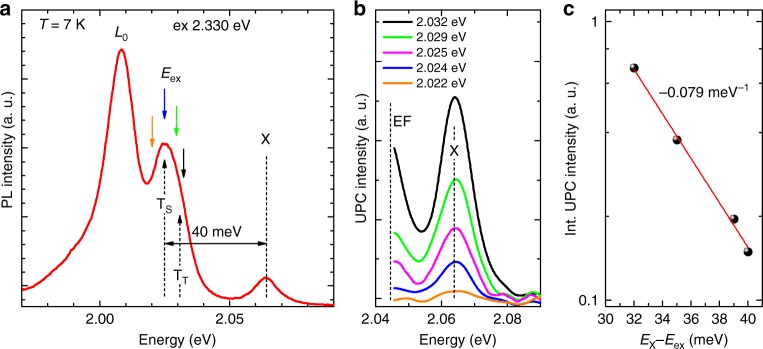
Upconversion photoemission process in monolayer WS_2_ at 7 K. **a** The PL spectrum recorded for WS_2_/hBN(136 nm)/SiO_2_/Si at 7 K. Black, green, blue and orange arrows point out excitation energies used in UPC experiment. **b** Examples of UPC spectra at 7 K excited with energies indicated by coloured arrows in **a**. **c** The dependence of the upconversion integrated intensity on the energy difference of the neutral exciton (X) and the excitation photon energy (E_X_–E_ex_)

It is important that the upconversion photoemission observed here is detected at room temperature. In order to gain further insight into the temperature dependence of the mechanism of the upconversion emission, we carried out temperature-dependent measurements of both the normal and upconverted photoluminescence of the WS_2_/SiO_2_/Si and WS_2_/hBN/SiO_2_/Si structures. The normal photoluminescence is excited at all temperatures with the same photon energy equals to 2.33 meV. The excitation energy of the upconversion photoluminescence is tuned to observe a detectable emission, and is varying from 1.959 eV to 2.033 eV at particular temperatures. Figure 5a shows a PL spectrum measured at 7 K for the WS_2_/hBN(136 nm)/SiO_2_/Si structure. The coloured arrows indicate excitation energies (E_ex_) of the upconversion photoluminescence presented in the Fig. 5b. The exciting photon energy required to achieve a detectable upconversion photoluminescence (gradual intensity growth just above the noise floor) at 7 K is found to exceed E_ex_ = 2.022 eV. Figure 5c shows the dependence of the integrated upconversion intensity (area under X peak integrated from 2.054 eV to 2.08 eV) on the energy difference E_X_–E_ex_ of the exciton X and the exciting photon energy E_X_. At 7 K the energy gain of upconversion emission amounts to about 42 meV, which is comparable with the energy difference between the X and T_S_ emission lines (spin singlet trion binding energy plus Fermi level energy^21^) and also nearly resonates with the energy of one optical phonon (A’_1_ or E’). This suggests that at low temperature the upconversion photoluminescence process is related to the coupling between the trion (T) and exciton (X) states mediated by one optical phonon. Similarly to the work of Jones et al.^11^, we find that the higher energy triplet trion state (T_T_) peak dominates the upconversion emission (see Fig. 5b). Moreover, for the energy difference E_X_–E_ex_ between 32 meV and 40 meV, the integrated upconversion intensity decreases with decreasing excitation photon energy, with the rate equal to −0.079 meV^−1^ (Fig. 5c), which is about three times higher (in absolute value) than the one estimated at 295 K under ambient condition.

**Fig. 6:**
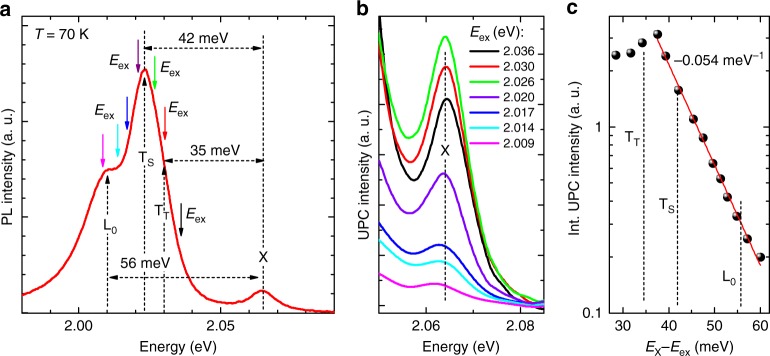
Upconversion photoemission process in monolayer WS_2_ at 70 K. **a** The PL spectrum recorded for WS_2_/hBN(136 nm)/SiO_2_/Si at 70 K. The coloured arrows point out excitation energies used in UPC experiment. **b** Examples of UPC spectra at 70 K excited with energies indicated by coloured arrows in **a**. **c** The dependence of the upconversion integrated intensity on the energy difference of the neutral exciton (X) and the excitation photon energy (E_X_ – E_ex_)

We now turn to the analysing a mechanism responsible for the upconversion photoluminescence at intermediate temperatures. Figure 6a shows the PL spectrum detected at 70 K. Coloured arrows indicate excitation energies of the upconversion photoluminescence spectra presented in Fig. 6b. At this temperature, the exciting photon energy required to achieve a detectable upconversion photoluminescence (gradual intensity growth just above the noise floor) is found to exceed E_ex_ = 2.009 eV. Following the upconversion photoluminescence spectra excited at different energies, presented in Fig. 6b, we see that with decreasing excitation energy from 2.036 to 2.026 eV, the intensity of the upconversion photoluminescence increases and achieves maximum at excitation with energy nearly equals to the energy of the trion in a spin singlet state T_s_ (green arrow in Fig. 6b). With further decreasing excitation energy, the upconversion photoluminescence decreases and is not detected at excitation energies below 2.009 eV. Figure 6c presents the dependence of the integrated intensity of upconversion photoluminescence on the energy difference E_X_–E_ex_ of the exciton X and the exciting photon energy E_X_. For the energy difference from 37 meV to 60 meV, the integrated upconversion photoluminescence intensity decreases with decreasing excitation photon energy with the rate equal to −0.054 meV^−1^, which is about two times higher and about one and half times lower (in absolute value) than those estimated under ambient at 295 and 70 K, respectively. Importantly, these results show that upconversion photoluminescence energy gain increases with increasing temperature. The energy gain of ~60 meV at 70 K suggests that more than one phonon is involved in the upconversion process.

To get further insight into the mechanism of photoluminescence at intermediate and high temperatures, we probe simultaneously the normal and upconversion photoluminescence spectra at temperatures from 20 K to 160 K. At all temperatures, the excitation of the normal photoluminescence is fixed at 2.33 eV, while the excitation energy of the upconversion photoluminescence is tuned in to maintain the constant energy separation between the excitation (E_ex_) and the exciton emission (E_X_). We have performed upconversion experiments for two different energy gains (E_X_–E_ex_) equal to 37 meV and 87 meV, respectively. Experiments are performed for two different WS_2_/hBN/SiO_2_/Si structures, which differ slightly in X emission intensity.

**Fig. 7:**
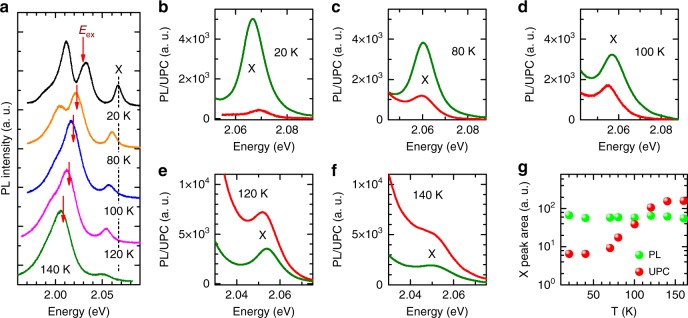
Temperature dependence of upconverted emission with the energy gain of 37 meV. **a** Schematic representation of the experiment: typical PL spectra with indicated by red arrows excitation energies used in UPC measurements at corresponding temperatures. **b**–**f** Examples of normal (green line) and upconversion (red line) photoluminescence spectra (green and red lines, respectively) recorded at 20 K, 80 K, 100 K, 120 K, 140 K, respectively. **g** The normal (green circles) and upconverted (red circles) excitonic photoluminescence integrated intensities as a function of temperature

In Figure 7, the study of the normal photoluminescence and upconversion photoluminescence with the energy gain of 37 meV at temperatures from 20 K to 160 K are presented. Figure 7a shows examples of the normal photoluminescence spectra at 20 K, 80 K, 100 K, 120 K, 140 K, respectively. For each temperature, the excitation energy, marked by red arrows, is tuned in to maintain the constant E_X_–E_ex_ energy separation of 37 meV, which nearly matches the energy of the trion (T) emission. Figure 7b–f compares the normal and upconversion photoluminescence spectra for WS_2_/hBN(136 nm)/SiO_2_/Si structure at temperatures 20 K, 80 K, 100 K, 120 K, 140 K, respectively. Figure 7g shows the normal (green circles) and upconversion (red circles) photoluminescence integrated intensities of the neutral exciton X as a function of temperature (from 20 K to 160 K). Both X emission intensities, at each temperature, are obtained by fitting of the PL/UPC spectra by Lorentz function in common energy range indicated in Fig. 7b–f, respectively. The normal and upconversion photoluminescence intensities present different trends with respect to temperature: upconversion photoluminescence intensity increases and exceeds standard photoluminescence intensity at 120 K, whereas normal photoluminescence remains nearly constant with increasing temperature. Among several investigated samples, both WS_2_/SiO_2_/Si and WS_2_/hBN/SiO_2_/Si, we find the same intensity trends of both the normal and upconversion photoluminescence. Here, the observed excitonic upconversion growth is governed by increasing phonon population^10^. It is also accompanied by simultaneous increase of the trion emission intensity with increasing temperature.

**Fig. 8:**
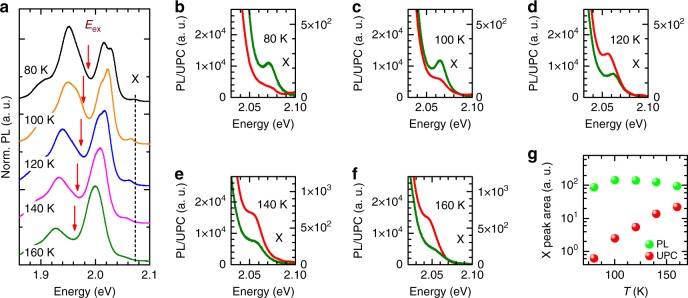
Temperature dependence of upconverted emission with the energy gain of 87 meV. **a** Schematic representation of the experiment: typical PL spectra with indicated by red arrows excitation energies used in UPC measurements at corresponding temperatures. **b**–**f** Examples of normal (green line, left vertical scale) and upconversion (red line, right vertical scale) photoluminescence spectra recorded at 80 K, 100 K, 120 K, 140 K, 160 K, respectively. **g** The normal (green circles) and upconverted (red circles) excitonic photoluminescence integrated intensities as a function of temperature from 80 K to 160 K

The study of the normal photoluminescence and upconversion photoluminescence with the energy gain of 87 meV of the WS_2_/hBN(115 nm)/SiO_2_/Si structure at temperatures from 80 K to 160 K are presented in Fig. 8. The schematic representation of the experiment is shown in Fig. 8a, which presents examples of the normal photoluminescence spectra at 80 K, 100 K, 120 K, 140 K, 160 K, respectively. At each temperature, the excitation energy of the upconversion photoluminescence, indicated by red arrows, is tuned in to keep the constant energy gain (E_X_–E_ex_) equal to 87 meV, which corresponds to the energy of the states from the trion tail and slightly exceeds the energy of localized excitons L. Figure 8b–f compares the normal and upconversion photoluminescence spectra of WS_2_/hBN(115 nm)/SiO_2_/Si structure at temperatures from 80 K to 160 K, respectively. As in the experiment with the lower energy gain (Fig. 7g), we find consistent intensity increase of X upconversion photoluminescence and nearly constant normal X photoluminescence in temperature range from 100 K to 160 K (see Fig. 8g). However, for the higher energy gain (87 meV), the intensity of X upconversion photoluminescence is relatively lower than the intensity of the normal X photoluminescence at each temperature. Here, the observed H excitonic upconversion growth at corresponding temperatures is again attributed to the increasing phonon population and is correlated with simultaneous increase of the trion emission. Additionally, this growth is accompanied by a gradual decrease of the localized excitons PL emission, also clearly observed in Fig. 8a or in Fig. 3e. These results indicate an efficient transfer between trion and exciton states mediated by resonant phonons. They also clearly confirm that in high temperature multi-phonon upconversion process the incident photon is rather in resonance with the states from the tail of the trion than in resonance with the exciton localized on the impurity.

**Fig. 9:**
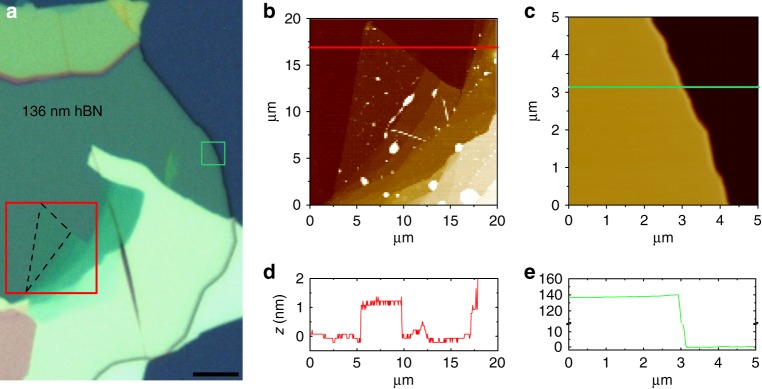
Optical microscope contrast and AFM image of WS_2_/hBN/SiO_2_/Si structure. **a** The optical microscope contrast image of WS_2_/hBN/SiO_2_/Si structure. The dashed black triangle shows the area corresponding to WS_2_ flake, whereas the red and green rectangles point out the areas corresponding to WS_2_/hBN/SiO_2_/Si and hBN/SiO_2_/Si, respectively. **b** The AFM image of the area indicated by the red rectangle in **a**. **c** The AFM image of the area indicated by the green rectangle in **a**. **d** The height profile along the red line overlaid on the image in **b**. **e** The height profile along the green line overlaid on the image in **c**

The example of the optical contrast microscope image and atomic force microscope (AFM) imaging of the typical, studied WS_2_/hBN/SiO_2_/Si heterostructure is presented in Fig. 9a–c.

Let us now discuss the efficiency of the upconversion photoluminescence process by means of the changes in the exciton emission intensity for different energy gains and gradual intensity descent rates. At 7 K, we estimate a change in the X line intensity of 0.632 decade (see Fig. 5c (40 meV – 32 meV) × 0.079 dec/meV). Then, at 70 K, we have a change in the X intensity of 1.2 decade (see Fig. 6c (60 meV – 37.5 meV) × 0.054), whereas at room temperature in ambient, we obtain a change in the X line intensity of 2.56 decade (see Fig. 2b (150 meV – 50 meV) × 0.0256). This simple analysis shows that at T = 295 K the upconversion photoluminescence intensity can be tuned by about two orders of magnitude over the mean energy gain of 100 meV.

## Discussion

To summarise, we demonstrate here a room temperature prominent upconversion photoluminescence process in a monolayer semiconductor WS_2_, with the energy gain up to 150 meV. We identified this process as transitions between trions dressed by phonons and free exciton complexes. We also show that upconversion photoluminescence energy gain significantly depends on the temperature and increases from 42 meV at 7 K to 150 meV at 295 K, indicating that high-temperature upconversion photoluminescence is indeed enabled by multi-phonon assisted transition. These results can be very promising for laser refrigeration, energy harvesting and optoelectronics with atomically thin materials.

## Methods

### Samples preparation

The monolayers of WS_2_ studied here were prepared by mechanical exfoliation of bulk crystals grown by chemical vapour transport technique (CVT). Prior to the crystal growth, the powdered compound was prepared from the elements (W: 99.99%; S: 99.999%) by reaction at *T* = 1000 °C for 10 days in evacuated quartz ampoules. The chemical transport was achieved with Br_2_ as a transport agent in the amount of about 5 mg/cm^3^.

We prepared WS_2_/hBN/SiO_2_/Si structures using hBN purchased from 2D Semiconductors. The WS_2_ crystals and hBN flakes with different thicknesses were mechanically exfoliated and then stacked using the deterministic all-dry stamping method, according to Castellanos-Gomez et al.^46^. We exfoliated hBN flake on a flexible PDMS Gel-Film stamp rigidly connected to a glass slide. Then the substrate and the stamp were placed under an optical microscope with XYZ stage attached. Application of long working distance objective enables us to locate and deterministically transfer selected hBN flakes on the substrate by carefully bringing the stamp in contact with the substrate. The same procedure has been repeated for monolayer WS_2_, which has been deterministically transferred on hBN flake. With that method, we prepared monolayer WS_2_ on hBN flakes with different thicknesses on Si substrates with 300 nm SiO_2_. Typical size of flakes exceeded 4 × 4 µm. Monolayer character of WS_2_ flakes was determined by their different optical contrast and has been confirmed by Raman and PL measurements on PDMS (to ensure the same experimental conditions) and, after transfer on target substrate, by atomic force microscopy (AFM) imaging (Fig. 9a–e).

### Experimental setup

The samples were mounted on a cold-finger of a non-vibrating closed circle cryostat, where temperature can be varied from 7 to 300 K. Photoluminescence was excited either by single mode lasers: the second harmonic 532 nm line of Nd: YAG and 632.8 nm line of a He–Ne, or by 610 – 675 nm lines of a DCM dye laser. The laser beam was focused on the sample under normal incidence using a 50× high resolution, long distance microscope objective (NA = 0.65). The diameter of excitation spot was equal to ~1 μm. The spectra were analysed with a 0.5 m focal length spectrometer and a 1200 lines/mm grating. A Peltier-cooled Si charge couple device was used as a detector. To eliminate the laser scattering light and phosphorescence of dye laser a set of short and long pass razor edge filters was used. The Raman scattering measurements were performed in the same set-up in backscattering geometry. The reflectivity contrast spectra were measured also in the same set-up, with 600 lines/mm grating and a filament lamp used as a light source.

## Data Availability

The data that support the findings of this study are available from the corresponding author on reasonable request.
